# Serum Exosomal miRNAs for Grading Hepatic Fibrosis Due to Schistosomiasis

**DOI:** 10.3390/ijms21103560

**Published:** 2020-05-18

**Authors:** Pengfei Cai, Yi Mu, Remigio M. Olveda, Allen G. Ross, David U. Olveda, Donald P. McManus

**Affiliations:** 1 Molecular Parasitology Laboratory, QIMR Berghofer Medical Research Institute, Brisbane 4006, Australia; Yi.Mu@qimrberghofer.edu.au; 2 Department of Health, Research Institute for Tropical Medicine, Manila 1781, Philippines; rolvedamd_ritm_doh@yahoo.com; 3 Menzies Health Institute Queensland, Griffith University, Gold Coast 4222, Australia; a.ross@griffith.edu.au (A.G.R.); dvdolveda@gmail.com (D.U.O.); 4 International Centre for Diarrhoeal Disease Research, Bangladesh (ICDDR, B), Dhaka 1212, Bangladesh

**Keywords:** schistosomiasis, *Schistosoma japonicum*, hepatic fibrosis, exosomal microRNAs, exomiRs, biomarker

## Abstract

Chronic infection with *Schistosoma japonicum* or *Schistosoma mansoni* results in hepatic fibrosis of the human host. The staging of fibrosis is crucial for prognosis and to determine the need for treatment of patients with schistosomiasis. This study aimed to determine whether there is a correlation between the levels of serum exosomal micro-ribonucleic acids (miRNAs) (exomiRs) and fibrosis progression in schistosomiasis. Reference gene (RG) validation was initially carried out for the analysis of serum exomiRs expression in staging liver fibrosis caused by schistosome infection. The expression levels of liver fibrosis-associated exomiRs in serum were determined in a murine schistosomiasis model and in a cohort of Filipino schistosomiasis japonica patients (*n* = 104) with different liver fibrosis grades. Of twelve RG candidates validated, miR-103a-3p and miR-425-5p were determined to be the most stable genes in the murine schistosomiasis model and subjects from the schistosomiasis-endemic area, respectively. The temporal expression profiles of nine fibrosis-associated serum exomiRs, as well as their correlations with the liver pathologies, were determined in C57BL/6 mice during *S. japonicum* infection. The serum levels of three exomiRs (miR-92a-3p, miR-146a-5p and miR-532-5p) were able to distinguish subjects with fibrosis grades I-III from those with no fibrosis, but only the serum level of exosomal miR-146a-5p showed potential for distinguishing patients with mild (grades 0–I) versus severe fibrosis (grades II–III). The current data imply that serum exomiRs can be a supplementary tool for grading liver fibrosis in hepatosplenic schistosomiasis with moderate accuracy.

## 1. Introduction

Schistosomiasis, a major public health problem, affects 230 million people worldwide [1,2]. Three species of schistosomes (trematode blood flukes), *Schistosoma haematobium*, *S. mansoni*, and *S. japonicum*, are the most clinically relevant. The infection of the latter two species results in the development of hepatopathology which is trigged by soluble egg antigen (SEA) released by eggs lodged in liver tissue. Several different types of immune cells (eosinophils, neutrophils, macrophages, and lymphocytes) are recruited to the periphery of deposited eggs and, along with resident liver cells, participate in the process [3,4]. Accumulating evidence shows that specific micro-ribonucleic acids (miRNAs) are dysregulated in particular cell types within the liver tissue, as well as in the circulatory system, during the development of liver fibrosis [5,6,7,8,9].

The gold standard for the diagnosis of liver fibrosis involves liver biopsy, which is highly invasive. In addition, serological diagnosis of liver fibrosis is currently challenging, due to the existing clinical biomarkers that have narrow applicability due to their lack of specificity and sensitivity [10]. The discovery of extracellular vesicles (EVs), small membranous nano-vesicles secreted by a variety of cell types, has evoked considerable interest for biomarker discovery, particularly in the field of cancer [11,12,13]. EVs include three main types; exosomes, microvesicles, and apoptotic bodies, based on their composition, biogenesis, and size [14]. EVs can transmit a wealth of bioactive cargos, such as DNA, messenger RNAs (mRNAs), miRNAs, proteins, and lipids, between cells, thus playing a vital role in cell–cell communication [15]. EVs stably circulate in many kinds of body fluids and protect the packaged nucleic acids from degradation. Serum/plasma EVs miRNAs also have been recently tested as being prospective predictors of virus-related hepatic fibrosis [7,8]. These two studies evaluated whether EV-derived miRNA signatures can provide a superior diagnostic performance than their counterparts isolated directly from serum/plasma in predicting the severity of liver fibrosis, yet contradictory results were obtained [7,8]. We recently measured the levels of circulating miRNAs directly isolated from serum to trace the progression of hepatic fibrosis during an infection caused by *S. japonicum* [9]. These investigations provide a key first step in identifying unique circulating host miRNAs associated with schistosome-associated liver fibrosis. We then considered it would be informative to determine whether measurement of serum exosomal miRNAs (exomiRs) can improve the ability to grade fibrosis in schistosomiasis. In this study, the widely used term ‘exosomes’ was used to refer to EVs, in general, unless otherwise specified.

Real-Time Quantitative Reverse Transcription PCR (qRT-PCR) is a powerful and widely used method to detect the levels of miRNAs in a variety of cell lines, tissues, and body fluids [16]. One prerequisite for valid analysis of qRT-PCR data is the normalization to the most stably expressed endogenous reference genes (RGs), particularly for assessment of gene expression in extracellular vesicles. To date, no reliable RGs have been identified that can be used to assess the levels of exosomal target miRNAs in serum/plasma during the development of liver fibrosis.

In light of our previous encouraging results [9], we report on further validation of serum exomiRs to improve on liver fibrosis grading in a mouse model of schistosomiasis and then in humans. C57BL/6 mice were initially employed to identify endogenous RGs in order to analyze serum exomiRs, and to determine the temporal expression profiles of nine exomiR candidates in serum using qRT-PCR assays during *S. japonicum* infection; the association between the expression levels of the investigated exomiRs with the severity of liver pathology was then determined. Then, the most stable endogenous RGs were identified within 12 exomiR candidates in a subset of cohort of individuals resident in a rural schistosomiasis-endemic area of the Philippines. Finally, 11 selected exomiR candidates were validated within serum samples from a cohort of schistosomiasis japonica patients with different grades of liver fibrosis. The study has further identified serum exomiRs that could serve as biomarkers for assessing the progression of hepatic fibrosis due to schistosomiasis in a rural endemic setting.

## 2. Results

### 2.1. Identification of Endogenous RGs for Serum exomiR Expression Analysis in C57BL/6 Mice during Schistosome Infection

To accurately quantify exosome derived miRNAs, the identification of appropriate endogenous reference genes (RGs) as calibrator is key. Based on the literature, 12 previously reported RGs tested in plasma/serum/cell extracellular vesicles/exosomes (Table 1) were selected for validation in a murine schistosomiasis model initially.

**Figure 1:**
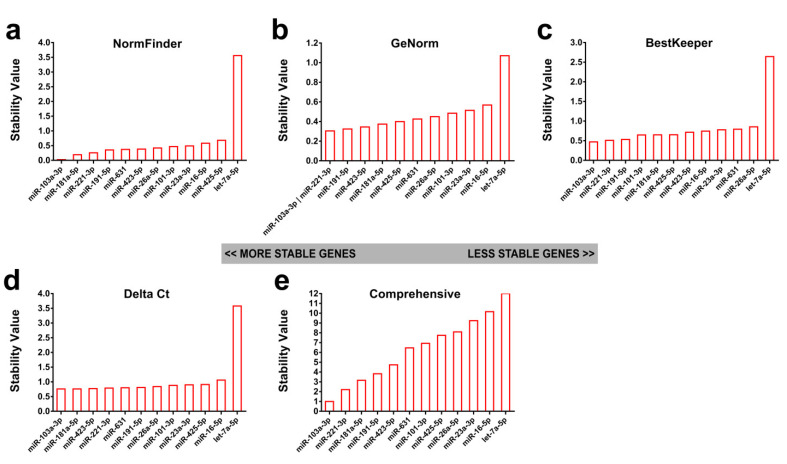
Identification of candidate reference genes (RGs) from Real-Time Quantitative Reverse Transcription PCR (qRT-PCR) quantification data of 12 exosomal microRNAs (exomiRs) in forty serum samples collected from C57BL/6 mice across the *S. japonicum* infection course (0 week p.i., *n* = 7; 4 weeks p.i., *n* = 7; 6 weeks p.i., *n* = 6; 7 weeks p.i., *n* = 6; 9 weeks p.i., *n* = 7; 11 weeks p.i., *n* = 7). Data were analyzed using (**a**) NormFinder, (**b**) GeNorm, (**c**) BestKeeper, and (**d**) Delta Ct. (**e**) A comprehensive ranking of candidate RGs was produced by RefFinder by integrating results of the above four algorithms.

After quantification of the RG candidates in serum exosome samples extracted from C57BL/6 mice during the infection course by RT-PCR, gene expression stability was analyzed using NormFinder, geNorm, BestKeeper, and Delta Ct to rank the RGs (Figure 1). NormFinder, BestKeeper, and Delta Ct identified miR-103a-3p as the most stable candidate, while geNorm identified both miR-103a-3p and miR-221-3p as the most stable candidates (Figure 1a-d). All algorithms identified let-7a-5p as the most unstable candidate. The geNorm ranked miR-103a-3p (0.305), miR-221-3p (0.305), and miR-191-5p (0.324) as being among the most stable RGs (Figure 1b). Using BestKeeper analysis, miR-103a-3p (0.471), miR-221-3p (0.509), and miR-191-5p (0.533) showed the lowest variation (Figure 1c). The geometric mean of all four above-mentioned methods was used to yield the final list of RG candidates, which identified miR-103a-3p (1.0), miR-221-3p (2.21) and miR-181a-5p (3.16) as being among the most stable genes, with miR-23a-3p, miR-16-5p, and let-7a-5p being the least stable species (Figure 1e).

### 2.2. Temporal Expression of Serum Exosomal miRNAs in C57BL/6 Mice during the Course of S. japonicum Infection

**Figure 2:**
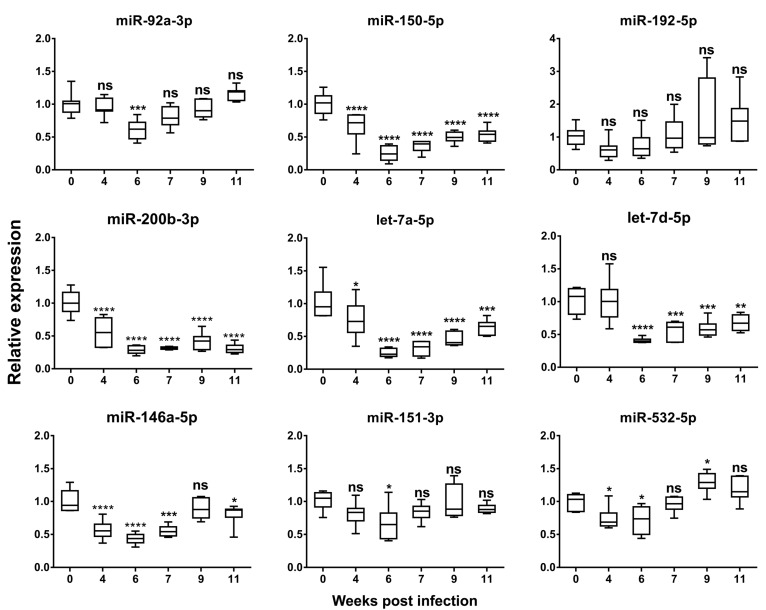
Temporal expression of serum exomiRs in C57BL/6 mice during *S. japonicum* infection. (Week 0, *n* = 7; week 4, *n* = 7; week 6, *n* = 6; week 7, *n* = 6; week 9, *n* = 7; week 11, *n* = 7). Boxes represent the interquartile range of the data. The hash marks above and below the boxes indicate the 90th and 10th percentiles for each group, respectively, while the lines across the boxes indicate the median values. *p* values were calculated using One-way ANOVA. (ns = no significant difference, * = *p* < 0.05, ** = *p* < 0.01, *** = *p* < 0.001, **** = *p* < 0.0001).

Based on previous studies [7,8,9,23,24], nine fibrosis-associated miRNA candidates were selected to test if their levels in serum exosomes were capable of predicting the grades of liver fibrosis during schistosomiasis progression (Table 2). Temporal expression of the nine exomiRs in serum was initially explored in C57BL/6 mice post-*S. japonicum-*infection with the most stable, exomiR miR-103a-3p, identified earlier used as an endogenous RG (Figure 2). Five of the nine exomiRs (miR-150-5p, let-7a-5p, let-7d-5p, miR-200b-3p, and miR-146a-5p) were significantly down-regulated during the infection course; the levels of three exomiRs, miR-150-5p, miR-200b-3p, and let-7a-5p, in serum were significantly down-regulated from 4 week p.i. and onwards, while the serum level of exomiR miR-146a-5p was also significantly down-regulated from 4 week p.i. and thereafter, except at 9 weeks p.i. Decreased expression in the level of serum exomiR let-7d-5p was observed from 6 weeks p.i. and subsequently. For the other exomiRs tested, the levels of miR-92a-3p and miR-151-3p were down-regulated only at 6 weeks p.i. The level of miR-532-5p in serum exosome was found significantly reduced at 4 and 6 weeks p.i., yet was up-regulated at 9 weeks p.i. No statistically significant alternation in the serum level of exosomal miR-192-5p was observed over the infection course.

### 2.3. Correlations of the Serum Levels of exomiRs with Liver Pathological Parameters in C57BL/6 Mice during S. japonicum Infection

**Figure 3:**
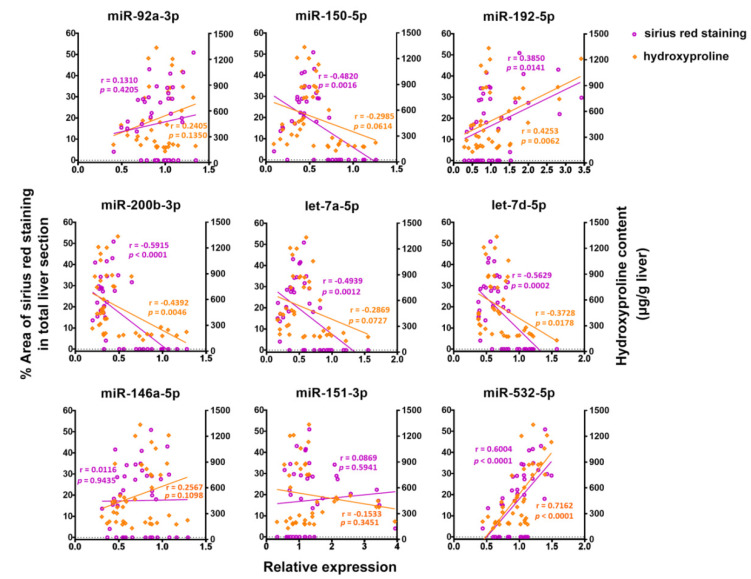
Correlations of serum levels of nine exomiRs with the intensities of liver fibrosis determined by sirius red staining and with liver hydroxyproline content in C57BL/6 mice during *S. japonicum* infection (Pearson’s *r*).

We then determined the correlations between the serum levels of the nine exomiRs and hepatic pathological parameters in C57BL/6 mice during the infection course (Table 3 and Figure 3). The levels of two exomiRs miR-151-3p and miR-192-5p showed a significant positive correlation with the serum levels of alanine aminotransferase (ALT) and aspartate aminotransferase (AST), while the levels of five exomiRs (miR-92a-3p, miR-150-5p, miR-200b-3p, let-7a-5p, and let-7d-5p) displayed a significant inverse correlation with the serum levels of liver enzymes (Table 3). The levels of two exomiRs (miR-192-5p and miR-532-5p) positively correlated with liver HP content, the intensity of liver fibrosis and the degree of granulomatous involvement in the liver; while the other two exomiRs (miR-200b-3p and let-7d-5p) negatively correlated with these liver pathological parameters (for miR-192-5p, *r* = 0.4253, 0.3850, and 0.3524, and *p* = 0.0062, 0.0141, and 0.0257, respectively; for miR-532-5p, *r* = 0.7162, 0.6004, and 0.5760, and *p* < 0.0001, < 0.0001, and = 0.0001, respectively; for miR-200b-3p, *r* = −0.4392, −0.5915, and −0.6210, and *p* = 0.0046, < 0.0001, and < 0.0001, respectively; for let-7d-5p, *r* = −0.3728, −0.5629, and −0.6063, and *p* = 0.0178, = 0.0002, and < 0.0001, respectively). The level of serum exosomal let-7a-5p significantly correlated with the intensity of hepatic fibrosis, the degree of granulomatous involvement and necrosis formed in the liver (*r* = −0.4939, −0.5529, and −0.3338, and *p* = 0.0012, 0.0002, and 0.0353, respectively). For the levels of miR-92a-3p and miR-146a-5p, the significant correlations were only observed with the level of liver necrosis (*r* = -0.3664 and −0.3625, and *p* = 0.0201 and 0.0215, respectively). The serum levels of another three exomiRs, miR-150-5p, miR-200b-3p, and miR-151-3p, also correlated with the intensity of liver necrosis in C57BL/6 mice during *S. japonicum* infection.

### 2.4. Identification of Endogenous RGs for Serum exomiR Expression Analysis in Schistosomiasis Patients with Different Liver Fibrosis Grading

**Figure 4:**
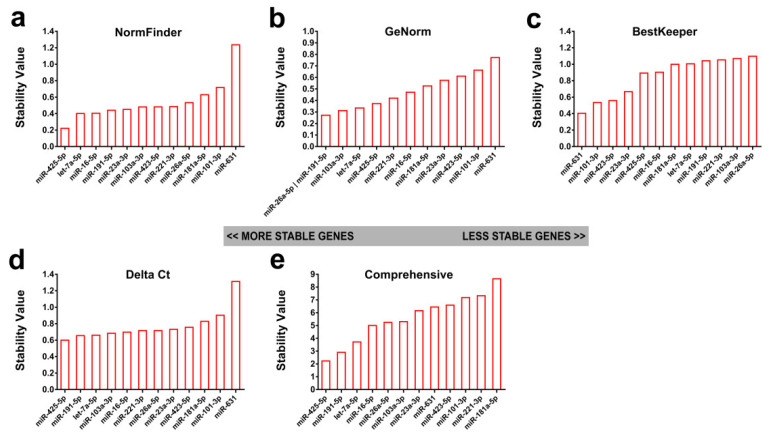
Identification of candidate RGs from qRT-PCR quantification data of 12 serum exomiRs in 42 subjects from a schistosomiasis-endemic area. Data were analyzed using (**a**) NormFinder, (**b**) GeNorm, (**c**) BestKeeper, and (**d**) Delta Ct. (**e**) A comprehensive ranking of candidate RGs was produced by RefFinder by integrating the results of the above four algorithms.

In order to identify suitable RGs for quantification of serum exomiRs in human schistosomiasis patients with different liver fibrosis grades, serum exosomal samples were extracted from 42 subjects resident in a schistosomiasis-endemic area. These subjects included 11, 11, 10, and 10 individuals with liver fibrosis grade 0, I, II, and III, respectively [25]. Then, the expression levels of the selected 12 candidate RGs in the serum exosomal samples were quantified by RT-PCR. Gene expression stability was then analyzed using the algorithms referred to above to rank the RGs (Figure 4). NormFinder identified miR-425-5p (0.222), let-7a-5p (0.402), and miR-16-5p (0.405) as the top three most stable candidates (Figure 4a). The geNorm ranked miR-26a-5p (0.272), miR-191-5p (0.272), and miR-103a-3p (0.312) as being among the most stable RGs (Figure 4b). Using BestKeeper analysis, miR-631 (0.404), miR-101-3p (0.535), and miR-423-5p (0.558) showed the lowest variation (Figure 4c). Using the Delta Ct method, miR-423-5p (0.6) was the most reliable, followed by miR-191-5p (0.656) and let-7a-5p (0.66) (Figure 4d). The geometric mean of all four of the methods was used to yield the final list of RG candidates, which identified miR-425-5p (2.236), miR-191-5p (2.913), and let-7a-5p (3.722) as being among the most stable genes, with miR-101-3p, miR-221-3p, and miR-181a-5p being the least stable species (Figure 4e).

### 2.5. Correlations of the Serum Levels of Eleven exomiRs with the Grades of Hepatic Fibrosis in a Cohort of Subjects from a Schistosomiasis Endemic Area

**Figure 5:**
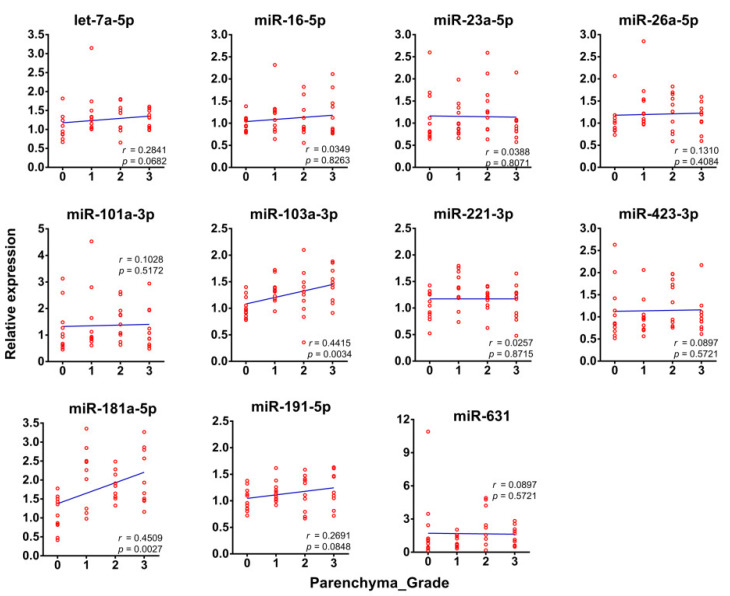
Correlations of the levels of the eleven exomiRs in serum (using the most stable exomiR miR-425 as reference) with hepatic fibrosis grades in the cohort subset employed for endogenous RGs identification (Spearman’s correlation coefficient).

Using the most stable exomiR (miR-425-5p) as reference, we analyzed the correlations of the serum levels of the other eleven RGs with hepatic fibrosis grades. A positive correlation was confirmed between the serum levels of the two exomiRs (miR-103a-3p and miR-181a-5p) with the grades of liver fibrosis within the 42 clinical samples tested for the identification of RGs (Figure 5).

**Figure 6:**
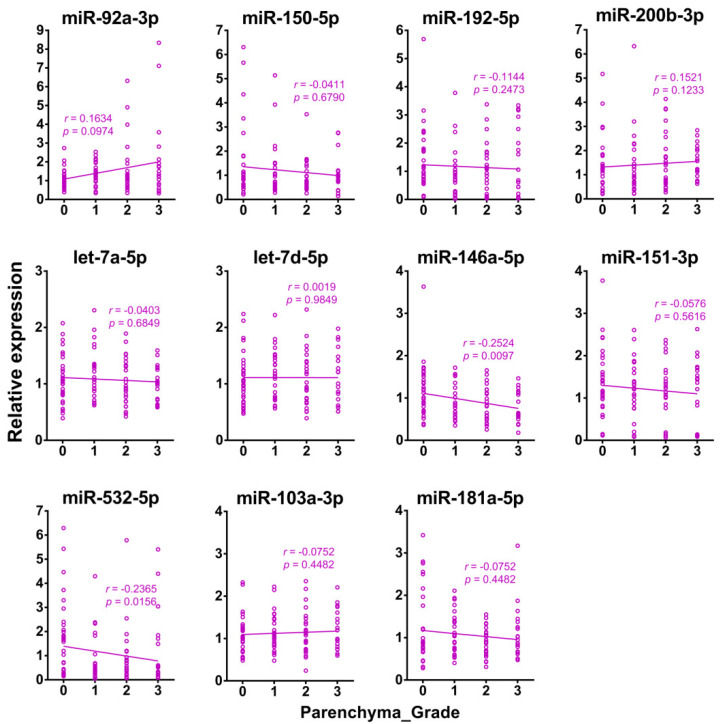
Correlations of the levels of eleven exomiRs in serum with hepatic fibrosis grades within the study cohort (Spearman’s *r*).

These two exomiRs along with the initially selected nine exomiRs and were further validated with a cohort of schistosomiasis japonica patients (*n* = 104) recruited from an endemic area of the Philippines (Table 4). The potential associations between the levels of the nine exomiR signatures in serum and the grades of liver fibrosis were then assessed. The serum levels of two exomiRs miR-146a-5p and miR-532-5p were inversely correlated with liver fibrosis grades (*r* = −0.2524 and −0.2365, and *p* = 0.0097 and 0.0156, respectively), whereas the others did not significantly correlate with hepatic fibrosis grading (Figure 6).

### 2.6. Serum-Derived exomiRs Can Discriminate Different Liver Fibrosis Grades

**Figure 7:**
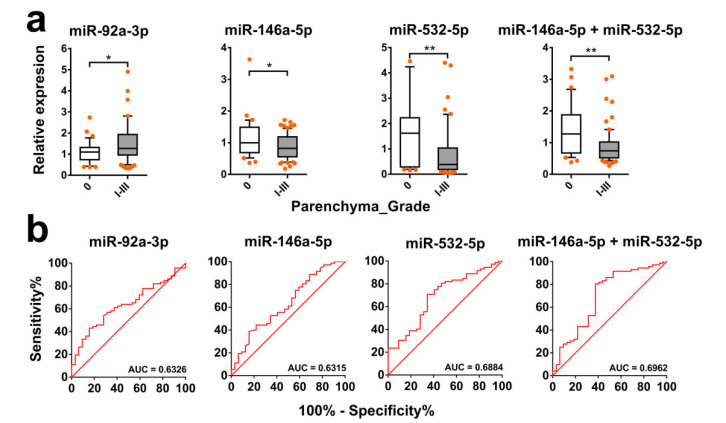
Discrimination of schistosomiasis hepatic fibrosis grades I–III vs. 0 by levels of exomiRs in serum. (**a**) The serum levels of exomiR candidates individually or in combination in subjects with (grades I–III) or without (grade 0) liver fibrosis. Boxes represent the interquartile range of the data with lines across the boxes indicating the median values. The hash marks above and below the boxes indicate the 90th and 10th percentiles, respectively. *p* values were calculated using a Mann–Whitney U test (* = *p* < 0.05, ** = *p* < 0.01). (**b**) The area under the curve (AUC) values were calculated using receiver operating characteristic (ROC) analysis to access capabilities of individual exomiR candidates or in combination in serum for discriminating subjects with stage I–III to those without liver fibrosis (grade 0).

**Figure 8:**
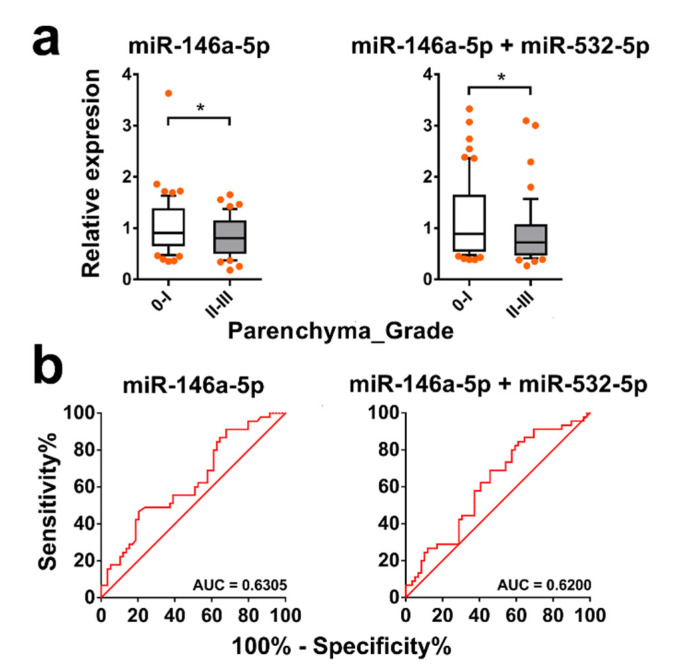
Discrimination of schistosomiasis hepatic fibrosis grades II–III vs. 0–I by serum levels of exomiRs. (**a**) The level of serum exosomal miR-146b-5p or a combination of exosomal miR-146a-5p and miR-532-5p in subjects with grades 0–I or II–III liver fibrosis. Boxes represent the interquartile range of the data with lines across the boxes indicating the median values. The hash marks above and below the boxes indicate the 90th and 10th percentiles, respectively. *p* values were calculated using a Mann–Whitney U test (* = *p* < 0.05). (**b**) The AUC values were calculated using ROC analysis to access capabilities of exomiR candidates individually or in combination in serum for discriminating subjects with severe fibrosis grades (II–III) from those without significant fibrosis (grades 0–I).

The levels of three exomiRs, miR-92a-3p, miR-146a-5p, and miR-532-5p, in serum exhibited a significant difference between subjects with fibrosis grades I-III compared with those without fibrosis (*p* = 0.0311, 0.0325, and 0.0020, respectively) (Figure 7a), while the levels of the other eight exomiRs did not show any significant difference between the two groups. The receiver operating characteristic (ROC) analysis for discriminating liver fibrosis grades I-III from grade 0 showed that the area under the curve (AUC) levels of the three exomiRs, miR-92a-3p, miR-146a-5p, and miR-532-5p, were 0.6326, 0.6315, and 0.6884, respectively (*p* = 0.0315, 0.0329, and 0.0023, respectively) (Figure 7b). The best diagnostic performance was obtained with a combination of exosomal miR-146a-5p and miR-532-5p, which showed an AUC value of 0.6962 (*p* = 0.0015) (Figure 7b). For discriminating liver fibrosis grades II–III from 0–I, we observed the serum level of exosomal miR-146a-5p or a combination of exosomal miR-146a-5p and miR-532-5p was significantly higher in individuals with mild fibrosis (grades 0–I) compared with those with severe disease (grades II–III) (*p* = 0.0227 and 0.0364, respectively) (Figure 8a), with an AUC of 0.6305 and 0.6200, respectively (*p* = 0.0231 and 0.0367, respectively) (Figure 8b). No exomiRs tested were able to discriminate subjects with liver fibrosis grades 0–II from those with grade III (data not shown).

## 3. Discussion

Liver fibrosis in schistosomiasis occurs during the development of a series of complex hepatopathologies, involving immune inflammation, granuloma formation, and liver damage [4], a scenario that may be more complicated than the etiology of other chronic liver diseases, such as those due to viral infections, alcoholic hepatitis, or non-alcoholic steatohepatitis. The current study aimed to determine whether the levels of exosome-derived miRNAs in serum are able to grade the intensities of liver fibrosis due to schistosomiasis. Identification of suitable RGs is a prerequisite for the quantification of miRNAs using qRT-PCR [27,28]. In this study, different exomiRs were identified as the most stable genes in a murine schistosomiasis model and within clinical samples. Across the infection course in C57BL/6 mice, exosomal miR-103a-3p was identified as the as the most stable RG, while exosomal miR-425-5p was determined as the most stable RG in patients infected with *S. japonicum*. Previous work recommended miR-103a-3p as a suitable RG for analysis of plasma miRNAs in a murine model with acetaminophen-induced hepatotoxicity [29]. Furthermore, miR-103a-3p was determined as the second stably expressed reference gene by the geNorm and NormFinder algorithms for analyzing serum exosomal miRNA expression in patients with hepatitis B or hepatocellular carcinoma [30]. On the other hand, miR-425-5p was identified as the most stable expressed miRNA in plasma of patients with vulvar intraepithelial neoplasia lesions and vulvar carcinoma [31] and in human plasma exosomes [21]. In the murine schistosomiasis model, the experimental time frame employed (4–11 weeks p.i.) represents an acute toxemic phase, which is characterized by complicated hepatic pathologies, including necrosis, inflammation and granuloma formation within a short period [32]. We noted in this study that the levels of several miRNA signatures in the circulatory system were not only associated with the severity of liver fibrosis but also with the extent of liver damage and the degree of granulomatous involvement (Table 3), features also evident in a previous study we undertook by directly measuring serum miRNAs in two murine models of schistosomiasis [9]. In contrast, schistosome infection in the human host tends to be chronic [9]. Thus, the animal model does not precisely represent the pathological status of human schistosomiasis, a feature that further highlights the importance of identification of suitable normalizers for the analysis of miRNA expression in extracellular vesicles in different disease models/conditions.

Comparative miRNAome analysis revealed different miRNA expression profiles in bovine sera and serum-derived exosomes [33]. Although it has been suggested that RNA packaging into extracellular vesicles/exosomes is a selective process, the precise loading mechanism for this remains elusive [34,35]. It is not surprising to observe the inconsistent performance of specific miRNA signatures derived from serum and serum exosomes as biomarkers for a particular disease. For example, Lambrecht et al. reported that levels of vesicle-associated miRNA-192, -200b-3p, -92a-3p, and -150-5p in plasma showed diagnostic power in discriminating early stage liver fibrosis, but this was not the case when these markers were directly measured in plasma [8]. In contrast, Matsuura et al. [7] showed that the plasma levels of let-7 family members significantly declined over the progression of fibrosis in patients with chronic hepatitis C, with an AUC value between 0.734 and 0.790; however, the detection power was reduced when targeting the exosome-derived let-7 family members. Similarly, we showed previously that the serum levels of miR-150-5p, let-7a-5p, and let-7d-5p in patients were able to discriminate mild from severe hepatic fibrosis caused by schistosome infection, with an AUC value of 0.6838, 0.6598, and 0.6270, respectively [9]. And, here, we found that the levels of these three miRNAs in serum exosomes were not capable of discriminating liver fibrosis grades 0 versus I–III, nor grades 0–1 versus II–III or grades 0–II versus III in the human subjects tested. Nevertheless, when compared with our previous study [9], similar trends were evident in the expression levels of several miRNAs (i.e., miR-150-5p, miR-192-5p, miR-200b-3p, let-7a-5p, let-7d-5p, and miR-146a-5p) in serum or in serum exosomes in C57BL/6 mice during *S. japonicum* infection.

It has been recently documented that miR-146a-5p participates in the biogenesis of fibrosis in a variety of tissues [36,37,38,39,40]. In the pathogenesis of nonalcoholic fibrosing steatohepatitis, it has been shown that miR-146a-5p was significantly down-regulated in activated hepatic stellate cells (HSCs) and overexpression of miR-146a-5p suppressed HSC activation, as well as extracellular matrix deposition [41]. During the development of liver fibrosis in a murine model of schistosomiasis, He et al. found that by targeting Signal Transducer And Activator Of Transcription 1 (STAT1), miR-146 suppressed the interferon (IFN)-γ-induced differentiation of macrophages to M1 cells [38]. In relation to the application of extracellular miR-146a-5p in staging liver fibrosis, Appourchaux et al. found that the serum level of miR-146a-5p was higher in chronic hepatitis C patients with advanced fibrosis and cirrhosis (F3–F4) than those with mild and moderate fibrosis (F1-F2); in contrast, no difference in the serum level of miR-146a-5p was observed between chronic hepatitis B patients with F3–F4 cirrhosis and those with F1–F2 [42]. In the current study, consistent with our previous study of direct measurement of serum miR-146a-5p [9], down-regulation of exosomal miR-146a-5p in the sera of C57BL/6 mice was observed over the course of *S. japonicum* infection, although no direct correlation between the serum level of exosomal miR-146a-5p and HP or HF was found. Furthermore, a negative correlation between the serum level of this miRNA and the grades of liver fibrosis was observed in the human cohort we investigated, which enables the discrimination of individuals with moderate to severe (grade II–III) liver fibrosis from those with mild (grade 0–I) disease. Nevertheless, there is still a concern that using a single miRNA/exomiR marker in the circulatory system for predicting a particular disease may incur a potential problem of non-specificity. For example, it has been shown recently that the level of serum exosomal miR-146a-5p declined significantly in systemic lupus erythematosus patients compared with healthy controls [43]. Accordingly, the use of a combination of multiple miRNA/exomiR signatures may improve diagnostic specificity.

Recent reports indicate that miR-532 family members are dysregulated in various tumors and are associated with carcinogenesis, thus having been considered as potential novel therapeutic interventions for the treatment of specific tumors [44,45,46]. Furthermore, Chen et al. found that serum EV miR-532-5p was significantly higher in control subjects versus F3/F4 fibrosis patients, using U6 as a reference gene [24]. Here, we showed that exosomal miR-532-5p inversely correlated with liver fibrosis grades in the human schistosomiasis cohort and was able to discriminate hepatic fibrosis I–III from those without fibrosis (AUC: 0.6884). However, in C57BL/6 during *S. japonicum* infection, a strong positive correlation was observed between the levels of serum exosomal miR-532-5p with the grades of liver fibrosis, which further echoed the different pathological severities in the murine schistosomiasis model, which usually represents a high dosage infection, compared with schistosome-infected patients.

Moderate diagnostic values were obtained in the grading of schistosomal liver fibrosis by detection of an individual exomiR or in combination. For discriminating individuals without liver fibrosis from those with grades I–III, the best diagnostic performance was obtained with the combination of serum exosomal miR-146a-5p and miR-532-5p (AUC: 0.6962, *p* = 0.0015) (Figure 7b). For discriminating mild from severe liver fibrosis, the diagnostic performance of exosomal miR-146a-5p in serum was not superior but comparable with that of miRNAs directly isolated from serum, i.e., AUCs of 0.6575 and 0.6305 were obtained for serum miR-146a-5p and serum exosomal miR-146a-5p, respectively. The following factors may contribute to this phenomenon: (i) It has been shown that re-infection with *S. japonicum* and co-parasitism with intestinal helminths and protozoa were common in the cohort subjects investigated [47,48], a feature which potentially might alter the host immune responses and further dysregulate the serum levels of the targeted exomiRs; (ii) as the study cohort individuals were from a rural area with rudimentary water sources and poor sanitation and hygiene [47], acute respiratory infections, diarrheal diseases, and other communicable diseases are likely to have had a considerable influence on the expression of the exomiRs investigated; (iii) only a limited number of exomiRs were tested in the current study, and it would be informative to identify more potential exomiRs specific for schistosomiasis fibrosis by profiling; (iv) importantly, not only the liver, but also other organs, i.e., the intestine, spleen and other ectopic sites (such as kidney and brain), are likely affected during hepatosplenic schistosomiasis [49]. Since serum exosomes can be released from all affected organs, the pathological alternation in multiple organs further likely changes the levels of exomiRs tested. Thus, assessment of miRNAs from sub-population exosomes, such as liver-derived [50] or, more specifically, HSCs-derived exosomes [51], may represent a promising pipeline to improve diagnostic power, as has been suggested for improving the performance of tumor diagnosis by tagging tumor-derived exomiRs in the blood circulation [52]. In this regard, membrane markers for liver-specific or HSC-specific exosomes need to be identified prior to applying this approach in the discovery of biomarkers for liver fibrosis or other liver diseases [53,54].

## 4. Materials and Methods

### 4.1. Ethics Statement

This study was approved by the QIMR Berghofer Medical Research Institute (QIMRB) Human Ethics Committee and the Ethics Committee of the Research Institute for Tropical Medicine (RITM), Manila, approved the study protocol in accordance with the Declaration of Helsinki (Institutional Review Board Number 2012-13-0, approved on 07/05/2012). Written informed consent was received from each study participant or from the legal guardians of those aged less than 15 years.

### 4.2. Animal Infection, Serum Collection, Histological Assessment, and Biochemical Analyses

Eight-week-old female C57BL/6 mice were divided into 6 groups and were percutaneously infected with 16 ± 2 of *S. japonicum* cercariae. Subsequently, mice were sacrificed at 0 (uninfected), 4, 6, 7, 9, and 11 weeks post-infection, and ~1 mL blood was collected from each mouse at each time point by cardiac puncture. The median lobe was collected from each mouse liver and subjected to histological assessment after formalin fixation [32]. Slides were scanned using the Aperio Slide Scanner (Aperio Technologies, Vista, CA, USA). Liver hydroxyproline (HP) content was assessed by a hydroxyproline colorimetric assay kit (Bioo Scientific, Austin, TX, USA). Serum alanine transaminase (ALT) and aspartate transaminase (AST) levels were tested using ALT and AST color endpoint assay kits (Bioo Scientific), respectively.

### 4.3. Study Cohort

The human cohort was recruited from 18 schistosomiasis-endemic but non-malaria-endemic barangays in the municipalities of Laoang and Palapag, Northern Samar, the Philippines in 2012. For more than over the last three decades, the area has had active schistosomiasis control programs, including a mass drug administration (MDA) program which commenced in 2008. Free annual treatment (Praziquantel 40 mg/kg in a single dose) is delivered to all individuals aged between 5–65 years in accordance with the Department of Health Administrative Order 2007–0015, but the compliance rate varies [55]. It is worthwhile to note that the *S. japonicum* reinfection rate in the area is also high [56]. Detailed information of this study cohort is described elsewhere as part of a hepatic morbidity study [25,47]. In brief, Kato–Katz thick smear stool examination was performed on each individual in the cohort using two stool specimens provided over the course of a week to determine infection status and intensity. Six Kato–Katz thick smears were prepared on microscope slides using standard 50 mg templates according to established protocols and examined under a light microscope by experienced technicians. All subjects in the cohort were assessed for liver fibrosis severity by ultrasound scan using a portable gray-scale ultrasonogram (SONOACE X1; Madison Co., Ltd., Seoul, South Korea) [25]. The parenchymal pattern of hepatic fibrosis was determined according to practical guidelines recommended by the WHO for ultrasonographic examination for schistosomiasis-related morbidity [57]. In the current study, 104 individuals (all negative for hepatitis B virus/hepatitis C virus infection) from the cohort were enrolled. For serum collection, ~10 mL blood was drawn from each subject enrolled; serum was subsequently separated by centrifugation, and stored at 2–8 °C. The serum samples were then transported to the Research Institute for Tropical Medicine (RITM), Manila, at a temperature of 4 °C and stored at −80 °C. Subsequently, sample aliquots were transported on dry ice to QIMRB, Australia.

### 4.4. Exosome Isolation, RNA Extraction, Polyadenylation, and Reverse Transcription (RT)

Serum exosomes were isolated using ExoQuick reagent (SBI System Biosciences, Palo Alto, CA, USA), according to the manufacturer’s protocol. Briefly, one hundred microliters of mouse or human serum was mixed with 31 µL of ExoQuick exosome precipitation solution. After incubation at 4 °C for 30min, the samples were then centrifuged at 1500× *g* for 30min. The supernatant was removed, and the exosome-rich pellet was re-suspended by adding 100 µL PBS. Subsequently, total RNA was extracted from the re-suspended samples using a Qiagen miRNeasy Mini Kit (Qiagen, Hilden, Germany), following the manufacturer’s instruction. RNA concentration was quantified using a Qubit MicroRNA Assay Kit (Thermo Fisher Scientific, Waltham, MA, USA). Polyadenylation and RT reactions were performed using the S-Poly(T) method in a one-step procedure by the combined use of two commercial kits: the Poly(A) Polymerase Tailing Kit (Lucigen, Middleton, WI, USA) and the TaqMan MicroRNA Reverse Transcription Kit (Thermo Fisher Scientific, Waltham, MA, USA) [32,58]. One ng RNA (5 μL) was used in each RT reaction, which was carried out under the following condition: 37 °C for 30 min, 42 °C for 30 min, and 85 °C for 5 min. RT products were stored at −20 °C prior to subsequent analysis. The RT primers used in this study are listed in Supplementary Tables S1–S3.

### 4.5. QRT-PCR for miRNA Quantification

Quantification of miRNAs was performed by qRT-PCR with the Applied Biosystems Quantstudio 5 Real-Time PCR System (Thermo Fisher Scientific, Waltham, MA, USA) according essentially to protocols described previously [32]. The assays were performed with the following cycling conditions: 50 °C, 2 min, 95 °C 10 min, followed by 40 cycles: 95 °C for 15 s, and 60 °C for 1 min. The expression levels of miRNAs were calculated by the 2^−ΔΔCt^ method with normalization to the reference genes identified in this study for analysis [32].

### 4.6. Reference Genes Identification

To identify RGs used for the analysis of exomiR expression in serum for predicting the severity of liver fibrosis, we evaluated two different data sets: (i) the temporal expression data of candidate RGs in a murine model during *S. japonicum* infection; (ii) the expression data of candidate RGs in serum samples of 42 subjects from a schistosomiasis-endemic area with different liver fibrosis grades. The relative expression stability of the candidates was analyzed using four computational programs (NormFinder [59], GeNorm [60], BestKeeper [61], and the comparative Delta Ct method [62]), which are collectively available at a user-friendly web-based RefFinder tool [63] (https://www.heartcure.com.au/reffinder). A recommended comprehensive ranking of candidate RGs was then generated by RefFinder based on the results of the four algorithms.

### 4.7. Statistical Analysis

For analysis of the temporal levels of serum exomiRs in C57BL/6 mice during *S. japonicum* infection, one-way ANOVA was used followed by the Holm-Sidak multiple comparison. Pearson’s correlation coefficient (*r*) was used for the determination of the correlations between continuous variables, while Spearman’s rank correlation coefficient (rho) was employed for the assessment of the correlations between continuous and categorical variables. The Mann–Whitney *U*-test was used for analysis of the ability of the levels of serum exomiRs in discriminating different schistosomiasis liver fibrosis grades. Receiver operating characteristic (ROC) curve analyses were performed, and the area under the curve (AUC) was calculated to evaluate the potency of using the serum exomiRs as biomarkers for discriminating hepatic fibrosis caused by schistosomiasis. A *p*-value < 0.05 was considered statistically significant. Statistical analysis was performed with GraphPad Prism v. 6.00 for Windows.

## 5. Conclusions

In summary, optimal reference genes were determined for studying exomiRs in a schistosomiasis murine model and a clinical cohort from a schistosomiasis endemic area, which may be of value for future research focused on serum/plasma exomiRs in fibrosis. We also identified serum exomiRs that are capable of grading hepatic fibrosis due to schistosomiasis japonica. The diagnostic performance of these exomiRs is moderate and, consequently, additional optimization steps, such as evaluating miRNAs in liver-derived or HSCs-derived exosomes [50], will be required to improve the overall diagnostic power using this approach.

## Figures and Tables

**Table 1:** Selected endogenous reference micro-ribonucleic acid (miRNA) candidates.

miRNA Name	Accession Number	Sequence	References
let-7a-5p	MIMAT0000062	UGAGGUAGUAGGUUGUAUAGUU	[17,18]
miR-16-5p	MIMAT0000069	UAGCAGCACGUAAAUAUUGGCG	[19]
miR-23a-3p	MIMAT0000078	AUCACAUUGCCAGGGAUUUCC	[19,20]
miR-26a-5p	MIMAT0000082	UUCAAGUAAUCCAGGAUAGGCU	[17,18,20]
miR-101-3p	MIMAT0000099	UACAGUACUGUGAUAACUGAA	[20]
miR-103a-3p	MIMAT0000101	AGCAGCAUUGUACAGGGCUAUGA	[17]
miR-221-3p	MIMAT0000278	AGCUACAUUGUCUGCUGGGUUUC	[17,18]
miR-423-5p	MIMAT0004748	UGAGGGGCAGAGAGCGAGACUUU	[21]
miR-425-5p	MIMAT0003393	AAUGACACGAUCACUCCCGUUGA	[21]
miR-181a-5p	MIMAT0000256	AACAUUCAACGCUGUCGGUGAGU	[18]
miR-191-5p	MIMAT0000440	CAACGGAAUCCCAAAAGCAGCUG	[18]
miR-631	MIMAT0003300	AGACCUGGCCCAGACCUCAGC	[22]

**Table 2:** Candidate miRNAs for grading liver fibrosis based on available literature.

miRNA	Accession Number	Sequence	References
miR-92a-3p	MIMAT0000092	UAUUGCACUUGUCCCGGCCUGU	[8,9]
miR-150-5p	MIMAT0000451	UCUCCCAACCCUUGUACCAGUG	[8,9]
miR-192-5p	MIMAT0000222	CUGACCUAUGAAUUGACAGCC	[8,9]
miR-200b-3p	MIMAT0000318	UAAUACUGCCUGGUAAUGAUGA	[8,9]
let-7a-5p	MIMAT0000062	UGAGGUAGUAGGUUGUAUAGUU	[7,9,23]
let-7d-5p	MIMAT0000065	AGAGGUAGUAGGUUGCAUAGUU	[7,9]
miR-146a-5p	MIMAT0000449	UGAGAACUGAAUUCCAUGGGUU	[9]
miR-151-3p	MIMAT0000757	CUAGACUGAAGCUCCUUGAGG	[24]
miR-532-5p	MIMAT0002888	CAUGCCUUGAGUGUAGGACCGU	[24]

**Table 3:** Correlations of the levels of exosomal miRNAs (exomiRs) in serum with liver pathological parameters in C57BL/6 mice during *S. japonicum* infection.

exomiR	AST		ALT		HP		HF^#^		GRA^#^		NEC^#^	
	*r*	*p*	*r*	*p*	*r*	*p*	*r*	*p*	*r*	*p*	*r*	*p*
miR-92a-3p	−0.4716	0.0021	−0.4126	0.0082	0.2405	0.1350	0.1310	0.4205	0.0695	0.6700	−0.3664	0.0201
miR-150-5p	−0.5213	0.0006	−0.6346	<0.0001	−0.2985	0.0614	−0.4820	0.0016	−0.5347	0.0004	−0.4191	0.0071
miR-192-5p	0.4035	0.0098	0.5393	0.0003	0.4253	0.0062	0.3850	0.0141	0.3524	0.0257	−0.0333	0.8386
miR-200b-3p	−0.4081	0.0089	−0.5874	<0.0001	−0.4392	0.0046	−0.5915	<0.0001	−0.6210	<0.0001	−0.3402	0.0317
let-7a-5p	−0.4960	0.0011	−0.5719	0.0001	−0.2869	0.0727	−0.4939	0.0012	−0.5529	0.0002	−0.3338	0.0353
let-7d-5p	−0.5382	0.0003	−0.6207	<0.0001	−0.3728	0.0178	−0.5629	0.0002	−0.6063	<0.0001	−0.2523	0.1163
miR-146a-5p	−0.2083	0.1972	−0.2196	0.1734	0.2567	0.1098	0.0116	0.9435	−0.0413	0.8002	−0.3625	0.0215
miR-151-3p	0.5395	0.0003	0.5304	0.0004	−0.1533	0.3451	0.0869	0.5941	0.1647	0.3099	0.3702	0.0187
miR-532-5p	−0.1504	0.3544	−0.1137	0.4849	0.7162	<0.0001	0.6004	<0.0001	0.5760	0.0001	−0.2977	0.0621

Correlations were analyzed by Pearson’s r (*n* = 40). Abbreviations: AST: aspartate aminotransferase; ALT: alanine aminotransferase; HP: hydroxyproline; HF: hepatic fibrosis; GRA: granuloma; NEC: necrosis. ^#^ Percentage of positive staining in total liver section.

**Table 4:** The infection status and intensity of the cohort stratified by fibrosis grade.

Fibrosis Grade	0 (*n* = 32)	I (*n* = 27)	II (*n* = 26)	III (*n* = 19)
M/F	12/20	22/5	21/6	19/0
Age (years)	36.69 ± 18.11	35.59 ± 15.94	46.19 ± 13.43	48.32 ± 13.25
Kato–Katz test (EPG range)	(0–220)	(0–423)	(0–633)	(0–747)
Negative (0)	27	17	15	6
Mild (1–99)	4	6	8	10
Moderate (100–399)	1	3	2	2
Heavy (>400)	0	1	1	1
Serological test (+/−)*	14/18	24/3	25/1	19/0

* Based on an ELISA assay detecting IgG antibodies against a combination of recombinant *S. japonicum* antigens (SjSAP4 plus Sj23-LHD) [26]. EPG: Egg per gram feces.

## Supplementary Materials

Supplementary materials can be found at https://www.mdpi.com/1422-0067/21/10/3560/s1. Table S1: Primers and probe used for optimal reference gene identification; Table S2: Primers and probe used for quantification of serum exosomal miRNA candidates for grading the intensities of liver fibrosis in a murine schistosomiasis model; Table S3: Primers and probe used for quantification of serum exosomal miRNA candidates for grading liver fibrosis in a human cohort from a schistosomiasis endemic area.
