# Supplementary material for: Serum Exosomal miRNAs for Grading Hepatic Fibrosis Due to Schistosomiasis
Source: Int J Mol Sci. 2020 May 18;21(10):3560. doi: 10.3390/ijms21103560 (PMC7278994; doi:10.3390/ijms21103560)
Supplement: Supplementary file 1 [file ijms-21-03560-s001.pdf]

Supplementary Information

Serum exosomal miRNAs for grading hepatic fibrosis due to schistosomiasis

Pengfei Cai, Yi Mu, Remigio M. Olveda, Allen G. Ross, David U. Olveda, Donald P. McManus

Supplementary Tables

Table S1. Primers and probe used for optimal reference gene identification.

	Target miRNA	Sequence (5'-3')
RT primer pool	let-7a-5p	CAGTGCAGGGTCCGAGGTCAGAGCCACCTGGGCAATTTTTTTTTTTAACTAT
	miR-16-5p	CAGTGCAGGGTCCGAGGTCAGAGCCACCTGGGCAATTTTTTTTTTTCGCCAA
	miR-23a-3p	CAGTGCAGGGTCCGAGGTCAGAGCCACCTGGGCAATTTTTTTTTTTGGAAAT
	miR-26a-5p	CAGTGCAGGGTCCGAGGTCAGAGCCACCTGGGCAATTTTTTTTTTTAGCCTA
	miR-101-3p	CAGTGCAGGGTCCGAGGTCAGAGCCACCTGGGCAATTTTTTTTTTTCAGTTA
	miR-103a-3p	CAGTGCAGGGTCCGAGGTCAGAGCCACCTGGGCAATTTTTTTTTTTCATAGC
	miR-221-3p	CAGTGCAGGGTCCGAGGTCAGAGCCACCTGGGCAATTTTTTTTTTTGAAACC
	miR-423-5p	CAGTGCAGGGTCCGAGGTCAGAGCCACCTGGGCAATTTTTTTTTTTAAAGTC
	miR-425-5p	CAGTGCAGGGTCCGAGGTCAGAGCCACCTGGGCAATTTTTTTTTTTCAACGG
	miR-181a-5p	CAGTGCAGGGTCCGAGGTCAGAGCCACCTGGGCAATTTTTTTTTTTACTCAC
	miR-191-5p	CAGTGCAGGGTCCGAGGTCAGAGCCACCTGGGCAATTTTTTTTTTTCAGCTG
	miR-631	CAGTGCAGGGTCCGAGGTCAGAGCCACCTGGGCAATTTTTTTTTTTGCTGAG
Forward primers	let-7a-5p	GGTGAGGTAGTAGGTTGTATAGTT
	miR-16-5p	GTAGCAGCACGTAAATATTGGC
	miR-23a-3p	GATCACATTGCCAGGGATTC
	miR-26a-5p	GTTCAAGTAATCCAGGATAGGC
	miR-101-3p	GGTACAGTACTGTGATAACTGAA
	miR-103a-3p	GAGCAGCATTGTACAGGGC
	miR-221-3p	GAGCTACATTGTCTGCTGGG
	miR-423-5p	TGAGGGGCAGAGAGCG
	miR-425-5p	GAATGACACGATCACTCCCG
	miR-181a-5p	GAACATTCAACGCTGTCGG
	miR-191-5p	GCAACGGAATCCCAAAAGC
	miR-631	AGACCTGGCCCAGACC
Universal reverse primer		CAGTGCAGGGTCCGAGGT
Universal double-quenched probe		56-FAM/CAGAGCCAC/ZEN/CTGGGCAATTT/3IABkFQ

Table S2. Primers and probe used for quantification of serum exosomal miRNA candidates for grading the intensities of liver fibrosis in a murine schistosomiasis model.

	Target miRNA	Sequence (5'-3')
RT primer pool	mmu-miR-92a-3p	CAGTGCAGGGTCCGAGGTCAGAGCCACCTGGGCAATTTTTTTTTTTCAGGCC
	mmu-miR-150-5p	CAGTGCAGGGTCCGAGGTCAGAGCCACCTGGGCAATTTTTTTTTTTCACTGG
	mmu-miR-192-5p	CAGTGCAGGGTCCGAGGTCAGAGCCACCTGGGCAATTTTTTTTTTTGGCTGT
	mmu-miR-let-7a-5p/let-7d-5p	CAGTGCAGGGTCCGAGGTCAGAGCCACCTGGGCAATTTTTTTTTTTAACYAT
	mmu-miR-200b-3p	CAGTGCAGGGTCCGAGGTCAGAGCCACCTGGGCAATTTTTTTTTTTCATCAT
	mmu-miR-146a-5p	CAGTGCAGGGTCCGAGGTCAGAGCCACCTGGGCAATTTTTTTTTTTAACCCA
	mmu-miR-151-3p	CAGTGCAGGGTCCGAGGTCAGAGCCACCTGGGCAATTTTTTTTTTTCCTCAA
	mmu-miR-532-5p	CAGTGCAGGGTCCGAGGTCAGAGCCACCTGGGCAATTTTTTTTTTTACGGTC
	mmu-miR-103a-3p	CAGTGCAGGGTCCGAGGTCAGAGCCACCTGGGCAATTTTTTTTTTTCATAGC
Forward primers	mmu-miR-92a-3p	GGTATTGCACTTGTCCCGG
	mmu-miR-150-5p	GTCTCCCAACCCTTGTACC
	mmu-miR-192-5p	GGCTGACCTATGAATTGACAGC
	mmu-let-7a-5p	GGTGAGGTAGTAGGTTGTATAGTT
	mmu-let-7d-5d	CCAGAGGTAGTAGGTTGCATAG
	mmu-miR-200b-3p	GGTAATACTGCCTGGTAATGATG
	mmu-miR-146a-5p	GGTGAGAACTGAATTCCATGGG
	mmu-miR-151-3p	TGCTAGACTGAAGCTCCTTGA
	mmu-miR-532-5p	TGCATGCCTTGAGTGTAGGA
	mmu-miR-103a-3p	GAGCAGCATTGTACAGGGC
Universal reverse primer		CAGTGCAGGGTCCGAGGT
Universal double-quenched probe		56-FAM/CAGAGCCAC/ZEN/CTGGGCAATTT/3IABkFQ

Table S3. Primers and probe used for quantification of serum exosomal miRNA candidates for grading liver fibrosis in a human cohort from a schistosomiasis endemic area.

	**Target miRNA**	**Sequence (5'-3')**
RT primer pool	Hsa-miR-150-5p	CAGTGCAGGGTCCGAGGTCAGAGCCACCTGGGCAATTTTTTTTTTTCACTGG
	Hsa-miR-92a-3p	CAGTGCAGGGTCCGAGGTCAGAGCCACCTGGGCAATTTTTTTTTTTACAGGC
	Hsa-miR-let-7a-5p/let-7d-5p	CAGTGCAGGGTCCGAGGTCAGAGCCACCTGGGCAATTTTTTTTTTTAACYAT
	Hsa-miR-200b-3p	CAGTGCAGGGTCCGAGGTCAGAGCCACCTGGGCAATTTTTTTTTTTCATCAT
	Has-miR-146a-5p	CAGTGCAGGGTCCGAGGTCAGAGCCACCTGGGCAATTTTTTTTTTTAACCCA
	Hsa-miR-181a-5p	CAGTGCAGGGTCCGAGGTCAGAGCCACCTGGGCAATTTTTTTTTTTACTCAC
	Hsa-miR-103a-3p	CAGTGCAGGGTCCGAGGTCAGAGCCACCTGGGCAATTTTTTTTTTTCATAGC
	Hsa-miR-425-5p	CAGTGCAGGGTCCGAGGTCAGAGCCACCTGGGCAATTTTTTTTTTTCAACGG
	Hsa-miR-191	CAGTGCAGGGTCCGAGGTCAGAGCCACCTGGGCAATTTTTTTTTTTCAGCTG
Forward primers	Hsa-miR-150-5p	GTCTCCCAACCCTTGTACC
	Hsa-miR-92a-3p	GGTATTGCACTTGTCCCGG
	Hsa-let-7a-5p	GGTGAGGTAGTAGGTTGTATAGTT
	Has-let-7d-5d	CCAGAGGTAGTAGGTTGCATAG
	Has-miR-200b-3p	GGTAATACTGCCTGGTAATGATG
	Has-miR-146a-5p	GGTGAGAACTGAATTCCATGGG
	Hsa-miR-181a-5p	GAACATTCAACGCTGTCGG
	Hsa-miR-103a-3p	GAGCAGCATTGTACAGGGC
	Hsa-miR-425-5p	GAATGACACGATCACTCCCG
	Hsa-miR-191	GCAACGGAATCCCAAAAGC
Universal reverse primer		CAGTGCAGGGTCCGAGGT
Universal double-quenched probe		56-FAM/CAGAGCCAC/ZEN/CTGGGCAATTT/3IABkFQ
